# Editorial: Acute liver injury and repair: cellular and molecular mechanisms

**DOI:** 10.3389/fcell.2024.1439921

**Published:** 2024-06-12

**Authors:** Xiaoqi Lin, Shuyu Shi, Lijian Chen, Po Gao, Liqun Yang

**Affiliations:** ^1^ Department of Anesthesiology, Renji Hospital, Shanghai Jiao Tong University School of Medicine, Shanghai, China; ^2^ Key Laboratory of Anesthesiology (Shanghai Jiao Tong University), Ministry of Education, Shanghai, China; ^3^ Department of Anesthesiology, The First Affiliated Hospital of Anhui Medical University, Hefei, China

**Keywords:** acute liver injury, liver repair, liver regeneration, acute liver failure, prognosis

Acute liver injury (ALI) and subsequent liver failure pose a significant burden to human health worldwide. Causes of acute liver injury vary, including viral hepatitis, exposure to hepatotoxins, immune system disorders and liver ischemia–reperfusion injury (LIRI) (Devarbhavi et al., 2023). With the prevalence of severe acute respiratory syndrome coronavirus 2 (SARS-CoV-2) pandemic, the morbidity of liver injury further increased (Phipps et al., 2020). Despite the unremitting efforts, effective treatment methods for acute liver failure (ALF) are still lacking. Yet it’s worth noting that the liver is the only visceral organ with regenerative ability. Therefore, it is of great importance to clarify the cellular and molecular mechanism of liver damage meanwhile enriching the perspective of liver regeneration mechanisms. The Research Topic titled “*Acute Liver Injury and Repair: Cellular and Molecular Mechanisms*” received several articles exploring the pathogenesis, prognosis indicators and tissue repair reaction in different liver diseases, providing new directions for the prevention and treatment of liver injury.

LIRI is one of the primary causes of early organ failure after transplantation, determining the prognosis of liver transplantation. Ito et al. summarized the roles of non-parenchymal cells in liver repair and regeneration after LIRI. Specifically, they put a spotlight on the response of liver macrophages, liver sinusoidal endothelial cells (LSECs), and hepatic stellate cells (HSCs) as well as the interactions between non-hematopoietic cell components and immune cell populations, which deepened the understanding about the pathology of liver IRI and laid foundation for investigating novel liver injury therapies.

The exploration of novel biomarkers for ALI and ALF is an important approach to improve the prognosis of liver disease and enhance patient survival. Several studies have shown that the expression of many cytokines and immune proteins correlates with the level of liver injury. Kumagai et al. elucidated that the expression level of glycoprotein non-metastatic melanoma protein B (GPNMB) in serum and liver was upregulated in patients with ALF and correlated with the severity of liver injury as well as the prognosis of ALI and ALF, suggesting that GPNMB may be a prognostic marker for patients with ALI and ALF. Zhu et al. found that serum cytokine/chemokine profiles were associated with 90-day prognosis in patients with HBV-ACLF, and established a composite immune-clinical prognostic model that included 2 immune indicators (IL-8 and CXCL2) and 2 clinical indicators (age and TBIL).

The liver is the only solid organ with significant regenerative capacity, and the influence of the immune system on liver repair and regeneration has been a focus of current research. Although many studies have reported changes in immune cells and cytokines during hepatic regenerative repair, the source of immune cells has not been fully elucidated (Hu et al., 2020; Di-Iacovo et al., 2023). The study by Elchaninov et al. demonstrated changes in the cytological structure and gene expression profiles of the spleen after 70% of hepatectomies and also found significant activation of the expression of protease inhibitor genes and migration of splenic monocytes-macrophages to the liver during the early stages of regeneration of the liver after hepatectomies.

In summary, the studies included in Research Topic focused on the in-depth investigation of the cellular and molecular mechanisms of acute liver injury and liver repair and regeneration processes, which provide the possibility of searching for prognostic markers of liver injury, identifying patients with high risk of death, and searching for novel therapeutic targets to develop new therapeutic strategies. Although the effects of the immune system and related cellular molecules on liver injury have been extensively studied, the role played by other organs and systems in this process remains unanswered. In particular, the central nervous system, as an important part of the neuro-endocrine-immune network, deserves further exploration for its influence on liver function.

## References

[B1] Devarbhavi H. Asrani S. K. Arab J. P. Nartey Y. A. Pose E. Kamath P. S. (2023). Global burden of liver disease: 2023 update. J. Hepatology 79, 516–537. 10.1016/j.jhep.2023.03.017 36990226

[B2] Di-Iacovo N. Pieroni S. Piobbico D. Castelli M. Scopetti D. Ferracchiato S. (2023). Liver regeneration and immunity: a tale to tell. Int. J. Mol. Sci. 24, 1176. 10.3390/ijms24021176 36674692 PMC9864482

[B3] Hu C. X. Wu Z. W. Li L. J. (2020). Mesenchymal stromal cells promote liver regeneration through regulation of immune cells. Int. J. Biol. Sci. 16, 893–903. 10.7150/ijbs.39725 32071558 PMC7019139

[B4] Phipps M. M. Barraza L. H. Lasota E. D. Sobieszczyk M. E. Pereira M. R. Zheng E. X. (2020). Acute liver injury in COVID-19: prevalence and association with clinical outcomes in a large US cohort. Hepatology 72, 807–817. 10.1002/hep.31404 32473607 PMC7300739

